# Xiaoyaosan Ameliorates Chronic Restraint Stress-Induced Depression-Like Phenotype by Suppressing A2AR Signaling in the Rat Striatum

**DOI:** 10.3389/fphar.2022.897436

**Published:** 2022-06-23

**Authors:** Xiaoxu Zhu, Qingyu Ma, Furong Yang, Xiaojuan Li, Yueyun Liu, Jianbei Chen, Lan Li, Man Chen, Xiaojuan Zou, Li Yan, Jiaxu Chen

**Affiliations:** ^1^ Guangzhou Key Laboratory of Formula-Pattern of Traditional Chinese Medicine, Formula-Pattern Research Center, School of Traditional Chinese Medicine, Jinan University, Guangzhou, China; ^2^ School of Basic Medical Sciences, Hubei University of Chinese Medicine, Wuhan, China; ^3^ Medical School, Hubei Minzu University, Enshi, China; ^4^ School of Traditional Chinese Medicine, Beijing University of Chinese Medicine, Beijing, China

**Keywords:** xiaoyaosan, depression, chronic restraint stress, adenosine receptor, microglia activation

## Abstract

Depression is a common mental disorder characterized by pessimism and world-weariness. In our previous study, we found that Xiaoyaosan (XYS) could have antidepressive effects, however the underlying mechanisms remain unclear. Several studies have shown that adenosine A (2 A) receptor (A2AR) in the brain is a key point in the treatment of depression. Our present study aimed to investigate the effects of XYS on A2AR signaling in the striatum of rats exposed to chronic restraint stress (CRS). Ninety-six male Sprague–Dawley rats were randomly divided into 8 groups (control, model, negative control, XYS, A2AR antagonist, A2AR antagonist + XYS, A2AR agonist, A2AR agonist + XYS). The rats in the model group, XYS group, A2AR antagonist group and A2AR antagonist + XYS group were subjected to CRS for 3 h a day. The XYS decoction [2.224 g/(kg·d)] was intragastrical administered by oral gavage to the rats in the negative control group, XYS group, A2AR antagonist + XYS group, and A2AR agonist + XYS group. The rats in the A2AR antagonist group and A2AR antagonist + XYS group were treated with SCH 58261 [0.05 mg/(kg·d)], and the rats in the A2AR agonist and A2AR agonist + XYS group were treated with CGS 21680 [0.1 mg/(kg·d)]. These procedures were performed for 21 consecutive days. Behavioral studies including the open field test, elevated plus maze test, sucrose preference test and forced swimming test, were performed to examine depression-like phenotypes. Then, the effects of XYS on CRS- or A2AR agonist-induced striatal subcellular damage, microglial activation and A2AR signaling changes in the striatum were examined. Here, we report that XYS ameliorates depression-like phenotypes (such as body weight loss as well as depression- and anxiety-like behaviors) and improves synaptic survival and growth in the stratum of the CRS rats. Moreover, XYS reduces A2AR activity and suppresses hyper-activation of striatal microglia. The tissue and cellular effects of XYS were similar to those of the known A2AR antagonists. In conclusion, XYS alleviates depression in the CRS rats via inhibiting A2AR in the striatum.

## Introduction

Depression is a common mental disorder characterized by depressed mood, loss of interest or happiness, a sense of guilt or self-negation, pessimism, poor sleep or appetite, fatigue, and inattention (Dubovsky et al., 2021). Approximately 350 million people of various ages suffer from depression worldwide (Summergrad, 2016). Patients with major depressive disorder show symptoms such as hallucinations, delusions and suicide attempts, which seriously limit their psychosocial function and reduce the quality of life of patients and their families (McCarron et al., 2021). Some studies indicated that chronic stress and psychological trauma leaded to nerve injure could induce depression (Williams et al., 2020).

Microglia are the resident immune cells of the brain. It has the function of immune surveillance in the central nervous system and can promote neural network pruning, regulate neural plasticity, and maintain and promote the smooth flow of neural pathways. Currently, microglia are gradually regarded as targets for the treatment of neurological and mental diseases (Biber et al., 2016). When neuromicroenvironmental changes are caused by local nerve injury (during neurodegenerative diseases) or social circumstance stress (stress) in the central nervous system, microglia can convert phenotypes. This process is called “microglia activation” (Dubbelaar et al., 2018). Microglia can be activated as immune-stimulatory (M1) or immunosuppressive (M2) phenotypes. There is ample evidence that microglial activation is involved in some mental disorders including depression (Dheen et al., 2007; Santiago et al., 2017). Therefore, depression can be caused by microglial lesion (Yirmiya et al., 2015).

As a metabolite of adenosine triphosphate (ATP) production, adenosine is an endogenous neuroprotective agent and neuro-regulator (Borroto-Escuela et al., 2018). The function of adenosine is mainly mediated by the adenosine receptor (AR). AR is very important for emotion regulation and is the main candidate target for regulating cognitive processes, enhancing sleep intention, and improving severe depression (Lazarus et al., 2019). AR directly regulates intrasynaptic information transmission and plasticity, which in turn affects various emotions, cognition, motor activity, neuroinflammation and cell death (Ohta and Sitkovsky, 2001). AR primarily includes the A1, A2A, A2B, and A3 receptors (A1R, A2AR, A2BR, and A3R). The main ARs involved in the regulation of neuroinflammation are adenosine A1 receptor (A1R) and adenosine A2A receptor (A2AR) (Peleli et al., 2017). At low concentrations, adenosine mainly activates A1R. Conversely, a high concentration of adenosine activates A2AR. A2AR can block heteromeric A1R through receptor–receptor allosteric trans-inhibition (Martí Navia et al., 2020). Caffeine, an A2AR antagonist, can effectively inhibit the activation of microglia, reduce neuroinflammation and exert an antidepressant effect through the A2AR/mitogen-activated protein kinase kinase (MEK)/extracellular signal-regulated kinase (ERK)/nuclear factor-κB (NF-κB) signaling pathway (Kaster et al., 2015; Mao et al., 2020).

The striatum is the origin of the basal ganglia and is the largest nucleus in it, where numerous A2AR heteroreceptor complexes are present. Some scholars believe that striatal morphology may be a biomarker of neurodegenerative diseases, or it may be the basis of internal phenotypes (Looi and Walterfang, 2013). Clinical studies have found that striatal abnormalities play a role in emotional and cognitive changes associated with severe depression (Furuyashiki and Deguchi, 2012). Experimental studies have found that chronic stress can cause behavioral changes, which is consistent with the morphological changes of the striatum subregion. Depression and other emotions are related to the abnormal activity of striatal neurons (Admon et al., 2017).

Xiaoyaosan (XYS) is one of the classic prescriptions for the treatment of mental disorders in traditional Chinese medicine. The experimental studies found that XYS has a significant antidepressant effect in the aspects of behavior, biochemistry, neurochemistry, intestinal microorganisms, gene expression profile and so on (Li et al., 2019; Ma et al., 2019). There are 121 bioactive compounds in XYS, which are related to 99 depression-related targets and participate in immune and inflammatory responses closely related to depression. UPLC-Q-TOF/MS analysis successfully identified all the key compounds of XYS as paeoniflorin, quercetin, luteolin, farnesin, aloe emodin, glyasperin C, and kaempferol, and the main compounds were flavonoids (Yuan et al., 2020). Our previous studies have found that XYS can improve depression-like behavior induced by chronic restraint stress (CRS) or chronic unpredictable mild stress, and its mechanism may include reducing the neuroinflammatory response (reduce IL-6, IL-1, NF-κB, TNF-α) (Zhu et al., 2021) and decreasing the level of glutamic acid (Glu) in the rat hippocampus (Zhou et al., 2021). In this study, we aimed to further explore whether the antidepressant effects of XYS are associated with A2AR signaling in the striatum.

## Methods and Materials

### Animals and Grouping

Ninety-six 8-week-old male Sprague–Dawley rats, weighing approximately 180–200 g, were purchased from Beijing Weitong Lihua Biotechnology Co., Ltd., Beijing. The rats were housed with 4 rats in plastic cages in SPF animal rooms: room temperature (25 ± 1)°C, relative humidity 30–40%, light and dark for 12 h (light 7:00:19:00, dark 19:00), free access to distilled water, and a regular rodent diet.

**FIGURE 1:**
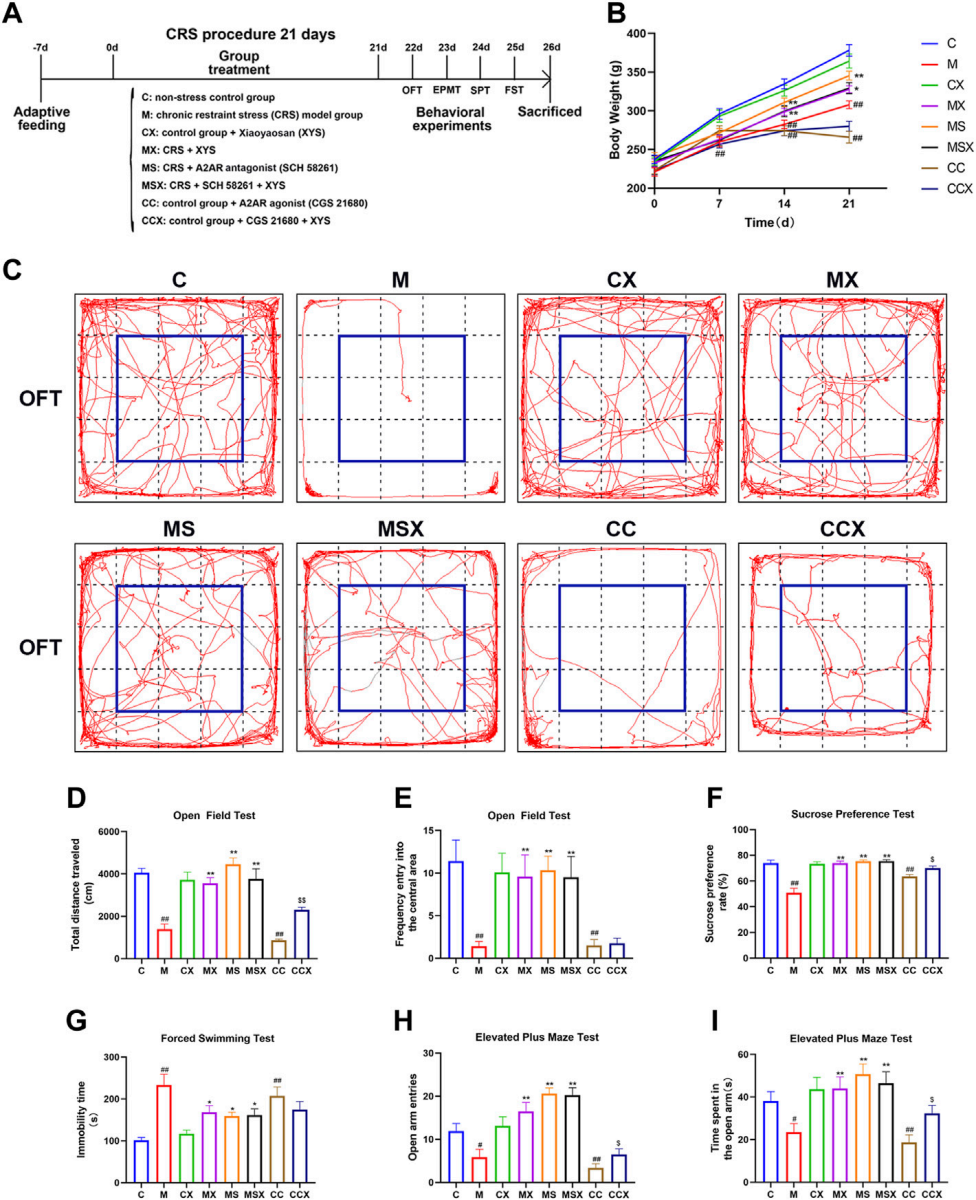
Effects of XYS on depression-like phenotypes. **(A)** Experimental flow chart. During the course of the experiment, the body weight of the rats was weighed and recorded every 7 days, and behavioral tests were performed on the rats in each group at the end of the experiment, including the OFT, EPMT, SPT, and FST. **(B)** Changes in body weight. **(C)** The representative trajectory map of rats in the OFT. **(D)** The total distance traveled in the OFT. **(E)** The frequency of entry into the central area in the OFT. **(F)** The sucrose preference rate in the SPT. **(G)** The immobility time in the FST. **(H)** The open arm entries in the EPMT. **(I)** The time spent in the open arm in the EPMT. Values are presented as the means ± SEM with 12 rats in each group. ^#^ *p* < 0.05 or ^##^ *p* < 0.01 versus the C group; ^*^ *p* < 0.05 or ^**^ *p* < 0.01 versus the M group; ^$^ *p* < 0.05 or ^$$^ *p* < 0.01 versus the CC group. XYS, Xiaoyaosan; OFT, open field test; EPMT, elevated plus maze test; SPT, sucrose preference test; FST, forced swimming test.

After 1 week of adaptive feeding, the rats were randomly divided into 8 groups: 1) control group (C, nonstress); 2) model group (M, CRS); 3) negative control group (CX, XYS); 4) XYS group (MX, CRS + XYS); 5) A2AR antagonist group (MS, CRS + SCH 58261); 6) A2AR antagonist + XYS group (MSX, CRS + SCH 58261 + XYS); 7) A2AR agonist group (CC, CGS 21680); and 8) A2AR agonist + XYS group (CCX, CGS 21680 + XYS). See Figure 1A for the flow chart of the experiment. In the whole process of the animal experiment, the welfare and ethical guidelines for experimental animals issued by the Animal Experimental Ethics Committee of Jinan University were strictly implemented, and the suffering of experimental animals was minimized.

### Chronic Restraint Stress Procedure

The rats were subjected to CRS as previously described (Zhu et al., 2019). The rats in the M group, MX group, MS group and MSX group were fixed to a special restraint rack for 3 h a day for 21 consecutive days. The rats in the other groups were freely dispersed in their respective feeding boxes, gently handled for 2–4 min, and returned back to the holding room, about 3 h later for 21 consecutive days.

### Xiaoyaosan Preparation and Drug Intervention

XYS powder was purchased from Jiuzhitang Group Co., Ltd.; the previously published literature (Hao et al., 2021) investigated the quality control of XYS using UPLC-Q-TOF/MS with the same formulation, batch number: 20190724. According to a previous study (Zhu et al., 2021), the XYS suspension (mixed with distilled water) was intragastrical administered by oral gavage to the rats in the CX group, MX group, MSX group, and CCX group at a dosage of 2.224 g/kg·d at 10 ml/kg body weight. The dosage was calculated according to the average body weight of an adult (60 kg/d). The rats in the other groups received 10 ml/kg distilled water by gavage. This procedure was performed 30 min before daily restraint stress and was performed for 21 consecutive days.

The A2AR antagonist SCH 58261 (M7282-01, Abmole, America) and A2AR agonist CGS 21680 (M2282-02, Abmole, America) were prepared with normal saline (NS, 0.9%) and injected intraperitoneally according to a body weight of 3 ml/kg. After 1 week of adaptation, the rats in the MS group and MSX group were treated with the A2AR antagonist SCH 58261, and the rats in the CC group and CCX group were treated with the A2AR agonist CGS 21680. The dosages were 0.05 mg/kg·d and 0.1 mg/kg·d, respectively (Pan and Chen, 2007; Costenla et al., 2011; Kaster et al., 2015; Huang et al., 2018). The rats in the other groups were given the same amount of 0.9% NS. This procedure was performed 30 min after daily restraint stress for 21 consecutive days.

### Behavioral Testing

Behavioral tests, including the open field test (OFT), elevated plus maze test (EPMT), sucrose preference test (SPT) and forced swimming test (FST) were performed. The behavioral tests were recorded by a camera, and the data were analyzed with a small animal behavior trajectory tracking and analysis system (NOLDUS EthoVision XT, Netherlands).

### Open Field Test

Rats were introduced to an open-field apparatus (100 × 100 × 40 cm) with a black background. The rats were placed in the middle of the box, and allowed rats to explore for 10 min. Their behavior was monitored by using a camera. The frequency of entry into the central square (60 × 60 cm) and total distance were calculated automatically (Schulz, 2018).

### Elevated Plus Maze Test

The EPMT apparatus consists of two open arms and two closed arms. Rats were placed in the central square and introduced into to the western arms to move freely for 5 min. The number of times the rats entered the open arm (two foreclaws must enter the arm) and the total time spent in the open arm within 5 min were calculated automatically by the software (Li et al., 2017).

### Sucrose Preference Test

Prior to the test, rats were housed in cages and trained to drink 1% sucrose solution for 24 h. Subsequently, rats were deprived of both food and water for the next 24 h. After that, the rats were tested in a single cage, and each cage was placed with a bottle of sucrose solution and a bottle of regular water. Rats were allowed to drink freely in 1 h, and each bottle was weighed before and after the test. The sucrose preference of the rats was calculated by the equation: sucrose preference (%) = [sucrose solution intake (g)/(sucrose solution intake (g) + regular water intake (g)] × 100% (Li et al., 2020).

### Forced Swimming Test

The rats were placed into plastic cylinder (50 cm deep, 20 cm in diameter) filled with water at 23–25°C up to a height of 30 cm from the base, and forced to swim for 6 min. The time of immobility behaviors (rat floating on the surface of the water, immobility of limbs, or slight body wiggling to maintain its balance) was calculated in the last 4 min (Ma et al., 2021).

### Detection of the Levels of Adenosine Triphosphate and Glutamic Acid in Striatum

On the 26^th^ day, all rats were anesthetized with an intraperitoneal injection of pentobarbital sodium (100 mg/kg), and the brain tissues were collected. The levels of ATP and Glu in the striatum were determined using commercial ELISA kits (ATP, Solarbio, Beijing, China; Glu, Nanjing Jiancheng Bioengineering Institute, Nanjing, China) according to the manufacturer’s instructions.

### Immunohistochemical Staining of Striatum

The rat brain specimens were fixed with 4% paraformaldehyde overnight, sectioned into 3-μm-thick paraffin sections. Before staining, the sections underwent dewaxing hydration and antigen retrieval. Soon after, the sections were blocked with 5% BSA for 1 h at room temperature. The primary antibodies (anti-rabbit IBA-1, ab178847, Abcam, United States, 1:8,000; anti-mouse A2AR, ab79714, Abcam, United States, 1:200) were used for incubation of sections overnight at 4°C. The sections were then incubated with secondary antibodies (donkey anti-rabbit IgG H&L, ab150073, Abcam, United States, 1:500; goat anti-mouse IgG H&L, E032410-01, EarthOx Life Sciences, United States, 1:500) for 1 h at room temperature. Finally, anti-fluorescence quenching agent with 4′-6-diamidino-2-phenylindole (DAPI) (#P0131; Beyotime, Shanghai, China) was used for mounting the sections. The stained sections were observed and the images were acquired by Axio Vert.A1 inverted microscope (Zeiss, Jena, Germany). The images were analyzed by measuring the mean optical density of the fluorescence with ImageJ software (NIH, United States) and normalized by the area.

### Golgi Staining

Golgi staining was performed by using a Hito Golgi-Cox OptimStainTM kit (Hito Biotec, Beijing, China). The right brain of rats were dissected out and placed in the impregnate solution, where they were stored in the dark for 24 h and were replaced with the same solution. After being stored for 2 weeks at room temperature in the dark, brains were transferred into solution-3, kept at 4 °C in the dark, replaced with solution-3 after 12 h, and stored at 4°C for 24 h. The coronal section of the brain tissue was cut into 160-μm-thick sections with a Leica vibrating microtome (VT1000S, Germany). The slices were then affixed to gelatin-coated slides, dried in the dark at room temperature for 12 h, stained with Solution-4 and Solution-5 according to the instructions, and sealed with neutral resin. The spine morphology was analyzed by Olympus laser scanning confocal microscope (FV3000, Japan). Spine densities were analyzed by cellSensDimension software. According to the classical classification of protruding morphology, dendritic spines are categorized into thin, mushroom, and stubby spines (Qiao et al., 2016). Spines are thin if the length is greater than the neck diameter and the diameters of the head and neck are similar. Spines are classified as mushrooms if the diameter of the head is greater than the diameter of the neck. Spines are considered stubby if the length and width are equal.

### Transmission Electron Microscopy Analysis

The specimens were removed from the striatum of the rat brains, which conformed to stereotaxic coordinates of interaural 10.20 mm and bregma 1.20 mm, fixed with fixative for TEM (G1102, Servicebio, Wuhan, China) and 1% OsO4 (Ted Pella Inc. CA, United States), then sectionalized to 100 nm, and finally dual stained with uranium acetate-lead citrate. Transmission electron microscopy (Hitachi, Tokyo, Japan) was used for observation of sealed sections as described previously (Peter et al., 2020). The observation focused on the sections of the microstructural alterations of the synaptic ultrastructure in the rat striatum. Excitatory asymmetric synapses were selected according to their typical structural organization, including the presynaptic terminal characterized by the presence of numerous synaptic vesicles, the synaptic cleft and a fuzzy electron-dense thickening of the post-synaptic membrane defining the post-synaptic density (PSD). PSD length and width were quantified using the ImageJ software (NIH, United States).

### Western Blotting Analysis

The expression of Na-K ATPase, A2AR, brain-derived neurotrophic factor (BDNF), Arg-1, iNOS, ERK and NF-κB p65 in the striatum was detected by WB analysis. Total striatal proteins were extracted using a total protein extraction kit (KTP300, Abbkine, China). Membrane proteins were extracted using a membrane protein extraction kit (P0033, Beyotime, China). Nuclear proteins were extracted using a nuclear protein and cytoplasm protein extraction kit (P0027, Beyotime, China). The total proteins were used for the detection of Na-K ATPase, BDNF, Arg-1, iNOS, and ERK, the membrane proteins were used for the detection of A2AR, while the nuclear proteins were used for the detection of NF-κB p65. The protein concentration was determined by a NanoDrop One (Thermo Scientific, United States). Then, an equal amount of protein (20 μg) from each sample was separated on 8% or 10% SDS–PAGE gels and transferred to polyvinylidene fluoride (PVDF) membranes. After transformation, the blot was placed into 5% defatted milk for 1–2 h at room temperature and then incubated with primary antibodies (anti-rabbit Na-K ATPase, #3010S, CST, United States, 1:1,000; anti-mouse A2AR, ab79714, Abcam, United States, 1:1,000; anti-rabbit BDNF, ab108319, Abcam, United States, 1:1,000; anti-rabbit Arg-1, #93668, CST, United States, 1:1,000; anti-mouse iNOS, SC-7271, Santa Cruz, United States, 1:500; anti-rabbit ERK1+ERK2, ab184699, Abcam, United States, 1:50,000; anti-rabbit phospho-ERK1+ERK2, #4370, CST, United States, 1:2,000; anti-rabbit NF-κB p65, GB11997, Servicebio, Wuhan, China, 1:500; anti-rabbit β-tubulin, #86298, CST, United States, 1:50,000; and anti-rabbit GAPDH, #5174, CST, United States, 1:1,000) overnight at 4°C, with β-tubulin and GAPDH as internal controls. The membrane was then incubated with a suitable secondary antibody for rabbits or mice for 1 h. Finally, the bands were observed with enhanced chemiluminescence reagent (Biorigin, Beijing, China), and the protein signals were analyzed by a ChemiDoc™ Imaging system (Bio–Rad, United States).

### Statistical Analysis

All the data in this study are expressed as the mean ± standard error of the mean (SEM). SPSS 25.0 software (Chicago, IL, United States) was used to perform these statistical analyses. The normality and variance uniformity of the experimental data were tested first, then repeated analysis of variance (ANOVA) was performed for the repeated measurement data, and one-way analysis of variance was used for the rest of the data. The least significant difference method was used for comparison. If the data were not normally distributed or the variance was not uniform, a nonparametric test with K independent samples was used. *p* < 0.05 was considered statistically significant. GraphPad Prism 8.0 Software (GraphPad Software Inc., San Diego, CA, United States) was used to perform plotting operations.

## Results

### Effects of Xiaoyaosan on Depression-Like Phenotype

To evaluate the effects of XYS on the depression-like phenotype, we measured body weight of rats and conducted a series of behavioral tests.

As shown in Figure 1B, there was no significant difference in the body weight of the rats among the 8 groups on the 0^th^ day (*p* > 0.05). However, compared with the C group, CRS and A2AR agonist significantly suppressed increases in body weight in the M group (from the 7^th^ day, *p* < 0.01) and CC group (from the 14^th^ day, *p* < 0.01), respectively. Compared with the M group, treatment with XYS and A2AR antagonists resulted in a significant increase in body weight gain in the MX group (from day 14, *p* < 0.01 or *p* < 0.05) and MS group (from day 14, *p* < 0.01). XYS also alleviated the trend of A2AR agonist-induced weight loss in rats, athough there was no significant difference (*p* > 0.05).

To investigate the effects of XYS on depression-like behaviors, the OFT, SPT and FST were performed. As shown in Figures 1C–G, compared with the rats in the C group, the rats subjected to CRS or A2AR agonist treatment displayed a lower total distance traveled (*p* < 0.01, *p* < 0.01) and lower frequency of entry into the central area (*p* < 0.01, *p* < 0.01) in the OFT, a lower sucrose preference rate (*p* < 0.01, *p* < 0.01) in the SPT, and a higher immobility time (*p* < 0.01, *p* < 0.01) in the FST. However, treatment with XYS or A2AR antagonist effectively reversed the changes caused by CRS (*p* < 0.05 or *p* < 0.01). XYS could also reverse the decrease in the total distance traveled in the OFT (*p* < 0.01) and sucrose preference rate in the SPT (*p* < 0.05) induced by A2AR agonists; however, there were no statistically significant differences in the frequency of entry into the central area in the OFT (*p* > 0.05) or the immobility time in the FST (*p* > 0.05) after XYS treatment.

To examine the effects of XYS on anxiety-like behaviors, the EPMT was observed. As shown in Figures 1H,I, the open arm entries (*p* < 0.05, *p* < 0.05) and time spent in the open arm (*p* < 0.01, *p* < 0.01) were lower in the M group and CC group than in the C group. Treatment with XYS or an A2AR antagonist reversed the changes induced by CRS (*p* < 0.01). XYS reversed the decrease in open arm entries (*p* < 0.05) and time spent in the open arms (*p* < 0.05) caused by A2AR agonists.

Taken together, our results indicate that treatment with XYS may mitigate CRS or the A2AR agonist-induced depression-like phenotype.

### Effects of Xiaoyaosan on the Microstructural Alterations of Dendritic Spine Morphology and Spine Density in the rat Striatum

In this study, we observed microstructural alterations in dendritic spine morphology in the striatum of rats.

**FIGURE 2:**
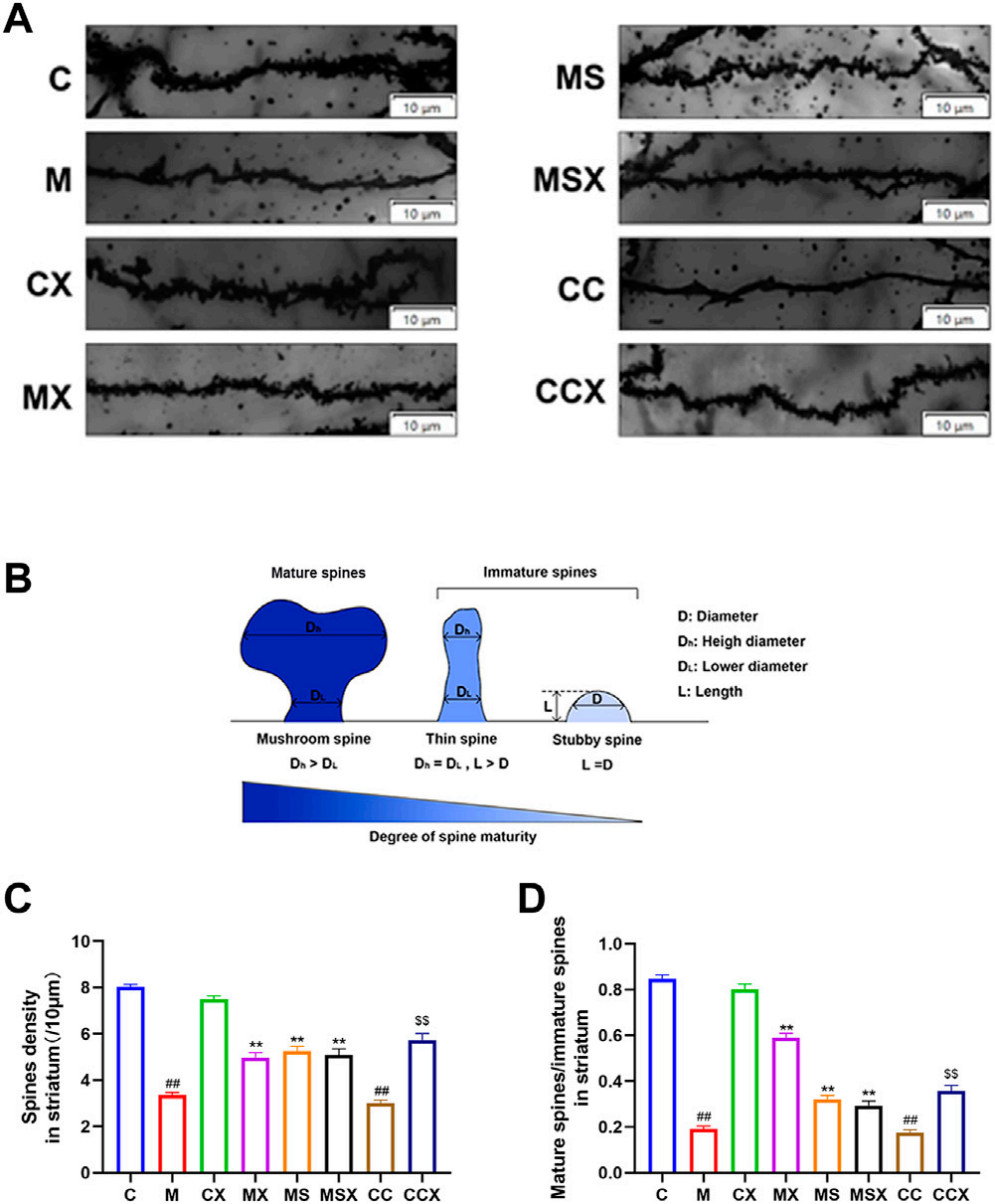
Effects of XYS on the microstructural alterations of dendritic spine morphology and spine density in the rat striatum. **(A)** Representative Golgi-stained dendritic spine image from the striatum region (scale bar = 10 μm). **(B)** Schematic map of mature and immature spines. **(C)** Spine density, 60 axons in each group. **(D)** The ratio of mature spines to immature spines; 60 axons in each group. The value is expressed as the means ± SEM, *n* = 3. ^##^ *p* < 0.01 versus the C group; ^**^ *p* < 0.01 versus the M group; ^$$^ *p* < 0.01 versus the CC group.

Figure 2A shows a representative Golgi-stained dendritic spine image from the striatum region. According to the classical classification of protruding morphology, as shown in Figure 2B, the spine density and ratio of mature/immature spines in the striatum region of rats was calculated.

The spine densities, as shown in Figure 2C, revealed that a significant reduction was present in the M group (*p* < 0.01) and the CC group (*p* < 0.01) compared with that observed in the C group. Spine densities in the MX group (*p* < 0.01), MS group (*p* < 0.01) and the MSX group (*p* < 0.01) were significantly greater than those in the M group. Spine densities in the CCX (*p* < 0.01) group were significantly greater than those in the CC group.

The ratio of mature/immature spines, as shown in Figure 2D, revealed a significant reduction in the M group (*p* < 0.01) and the CC group (*p* < 0.01) compared with the C group. XYS or A2AR antagonist increased the low ratio of mature/immature spines caused by CRS (*p* < 0.01, *p* < 0.01). XYS also increased the low ratio of mature/immature spines caused by A2AR agonist (*p* < 0.01).

In summary, XYS ameliorated the microstructural alterations in dendritic spine morphology and spine density in the rat striatum induced by CRS and A2AR agonists.

### Effects of Xiaoyaosan on the Synaptic Ultrastructure in rat Striatum

**FIGURE 3:**
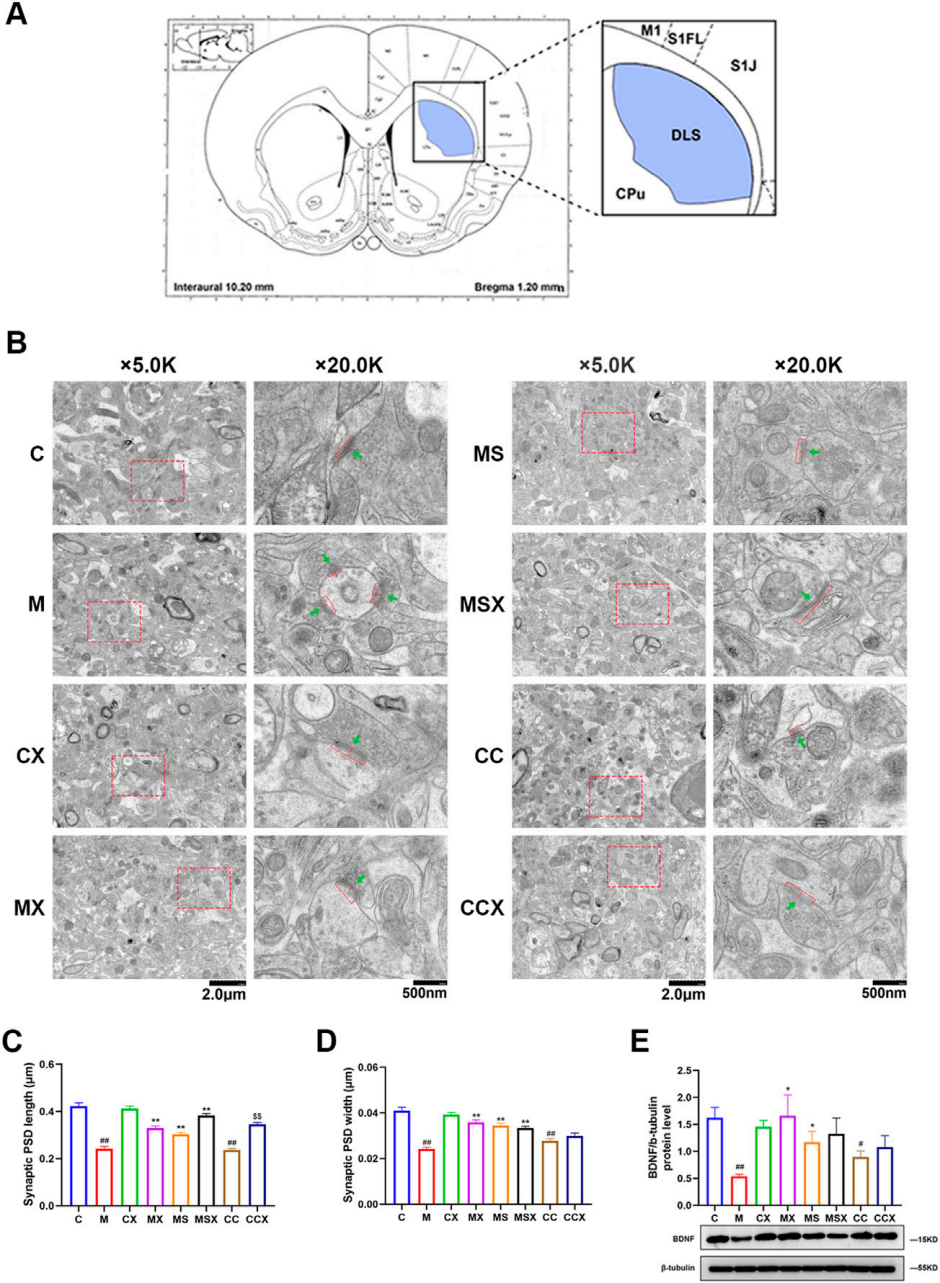
Effects of XYS on the synaptic ultrastructure in the rat striatum. **(A)** Electron microscope section and photographic site diagram of the striatum. Synapses were observed under transmission electron microscopy. **(B)** Representative microphotographs of the rat striatum. The first column of microphotographs was taken at low magnification (scale = 2 μm, 5,000× magnification) under the electron microscope, and the second column of microphotographs was taken at high magnification (scale = 500 nm, 20,000× magnification) under the electron microscope. Red bars and red arrows in lower panels indicate PSD length and PSD width, respectively. Green arrows indicate synaptic vesicles. **(C)** Synaptic PSD length in the rat striatum, 60 synapses in each group. **(D)** Synaptic PSD width in the rat striatum, 60 synapses in each group. **(E)** Expression of BDNF protein in the rat striatum. The value is expressed as the means ± SEM, *n* = 3. ^#^ *p* < 0.05 or ^##^ *p* < 0.01 versus the C group; ^*^ *p* < 0.05 or ^**^ *p* < 0.01 versus the M group; ^$$^ *p* < 0.01 versus the CC group. PSD, post-synaptic density; BDNF, brain-derived neurotrophic factor.

The rat striatum was sectioned and photographed under an electron microscope according to Figure 3A. Figure 3B is a representative microphotograph of the striatum under a transmission electron microscope.

We performed an ultra-structure analysis of PSD by transmission electron microscopy. As shown in Figures 3C,D, compared with the rats in the C group, both PSD length and width in the striatum region of rats in the M group (*p* < 0.01, *p* < 0.01) and CC group (*p* < 0.01, *p* < 0.01) were significantly reduced, whereas treatment with XYS (*p* < 0.01, *p* < 0.01) or A2AR antagonist (*p* < 0.01, *p* < 0.01) effectively reversed the changes caused by CRS. XYS also reversed the reduce in the PSD length in the striatum induced by A2AR agonists (*p* < 0.01). However, there were no statistically significant differences in the PSD width induced by A2AR agonists after XYS treatment (*p* > 0.05).

As shown in Figure 3E, compared with the C group, the protein expression level of BDNF in the rat striatum was decreased in the M group (*p* < 0.01). XYS or the A2AR antagonist ameliorated the decrease in synapse-associated proteins caused by CRS (*p* < 0.01). The A2AR agonist decreased the protein expression of BDNF compared with the C group (*p* < 0.05). XYS down-regulated the A2AR agonist-induced reduction in BDNF protein expression, although the difference was not statistically significant (*p* > 0.05).

These results suggest that XYS can reduce the damage to the synaptic ultrastructure in the rat striatum induced by CRS and plays a protective role in the synaptic ultrastructure.

### Effects of Xiaoyaosan on the Activation of Microglia in rat Striatum

In this study, immunofluorescence assays and WB were used to detect the activation of microglia in the rat striatum.

**FIGURE 4:**
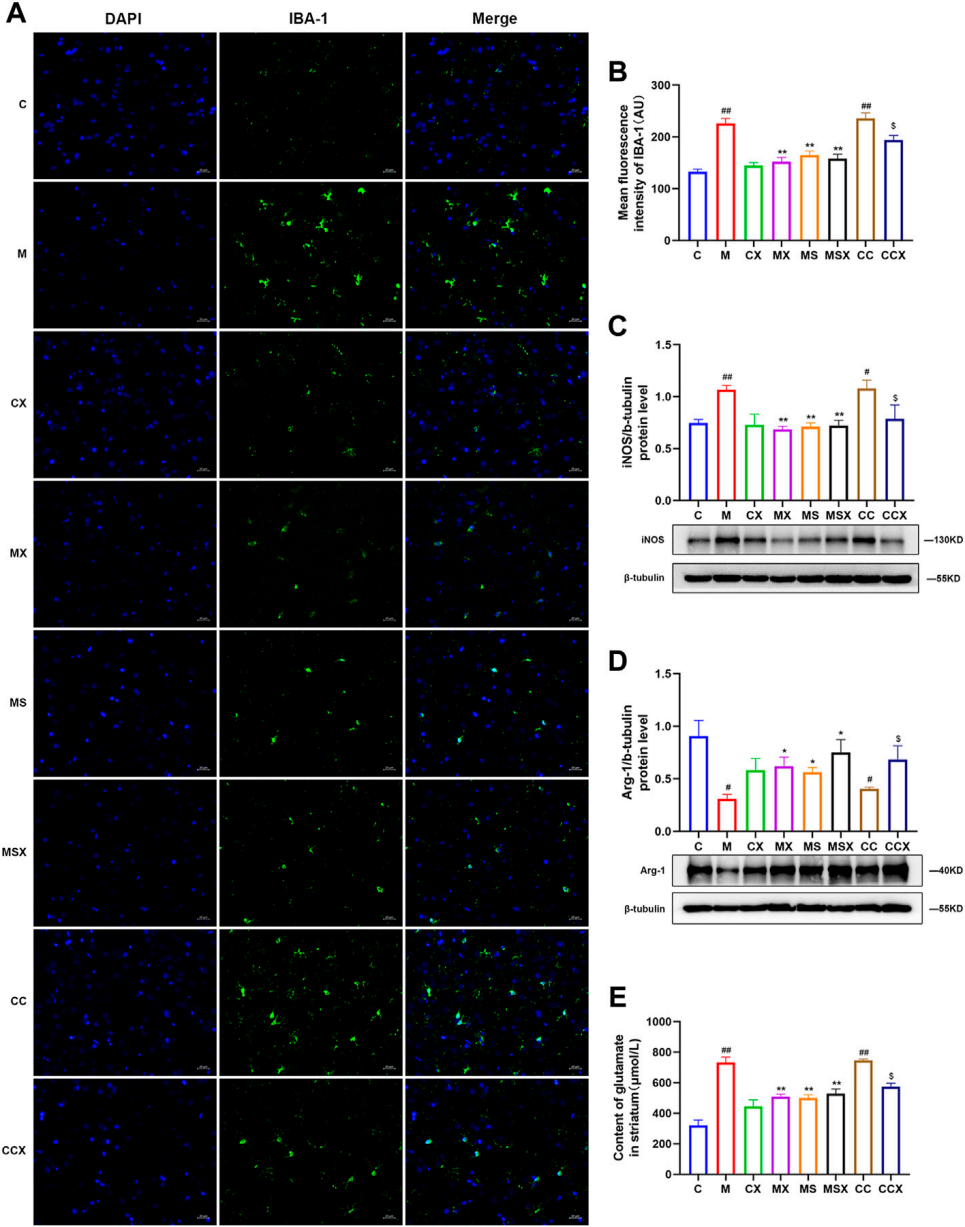
Effects of XYS on microglial activation in the rat striatum. **(A)** Representative micrographs of immunofluorescence staining for the microglial activation marker IBA-1 in the rat striatum (scale = 20 μm, 400× magnification). **(B)** Mean fluorescence density of IBA-1. **(C)** The protein expression of iNOS in the rat striatum. **(D)** Arg-1 protein expression in the rat striatum. **(E)** The content of Glu in the rat striatum. The value is expressed as the mean ± SEM, *n* = 3. ^#^ *p* < 0.05 or ^##^ *p* < 0.01 versus the C group; ^*^ *p* < 0.05 or ^**^ *p* < 0.01 versus the M group; ^$^ *p* < 0.05 versus the CC group. IBA-1, ionized calcium-binding protein-1; iNOS, inducible nitric oxide synthase; Arg-1, arginase-1; Glu, glutamate.

As shown in Figures 4A,B, the fluorescence intensity of the microglial activation marker IBA-1 was significantly increased in the M group (*p* < 0.01) and CC group (*p* < 0.01) compared with the C group. Treatment with XYS (*p* < 0.01) or an A2AR antagonist (*p* < 0.01) reversed the changes caused by CRS. XYS also reversed the increase in IBA-1 expression and microglial activation caused by the A2AR agonist (*p* < 0.05).

As shown in Figures 4C,D, compared to the C group, the protein expression of the M1-associated marker iNOS (*p* < 0.01, *p* < 0.05) was higher and that of the M2-associated marker Arg-1 (*p* < 0.05, *p* < 0.05) was lower in the M group and CC group. XYS or SCH 58261 reversed the changes caused by CRS (*p* < 0.05 or *p* < 0.01). XYS also reversed the changes caused by the A2AR agonist (*p* < 0.05).

As shown in Figure 4E, the Glu levels in the striatum of rats were measured, and the results showed that CRS (*p* < 0.01) and the A2AR agonist (*p* < 0.01) increased the secretion of Glu in the striatum compared with the C group. XYS (*p* < 0.01) or SCH 58261 (*p* < 0.01) decreased the Glu levels in the striatum compared with the M group. XYS also decreased the Glu levels compared with those in the CC group (*p* < 0.05).

In summary, the above results confirmed that treatment with XYS may alleviate CRS or A2AR agonist-induced overactivation of microglia in the rat striatum.

### Effects of Xiaoyaosan on the Striatal Adenosine A2A Receptor-Extracellular Signal-Regulated Kinase-Nuclear Factor-κB Pathway

The expression of the A2AR-ERK-NF-κB pathway was detected by immunofluorescence assay and WB analysis.

**FIGURE 5:**
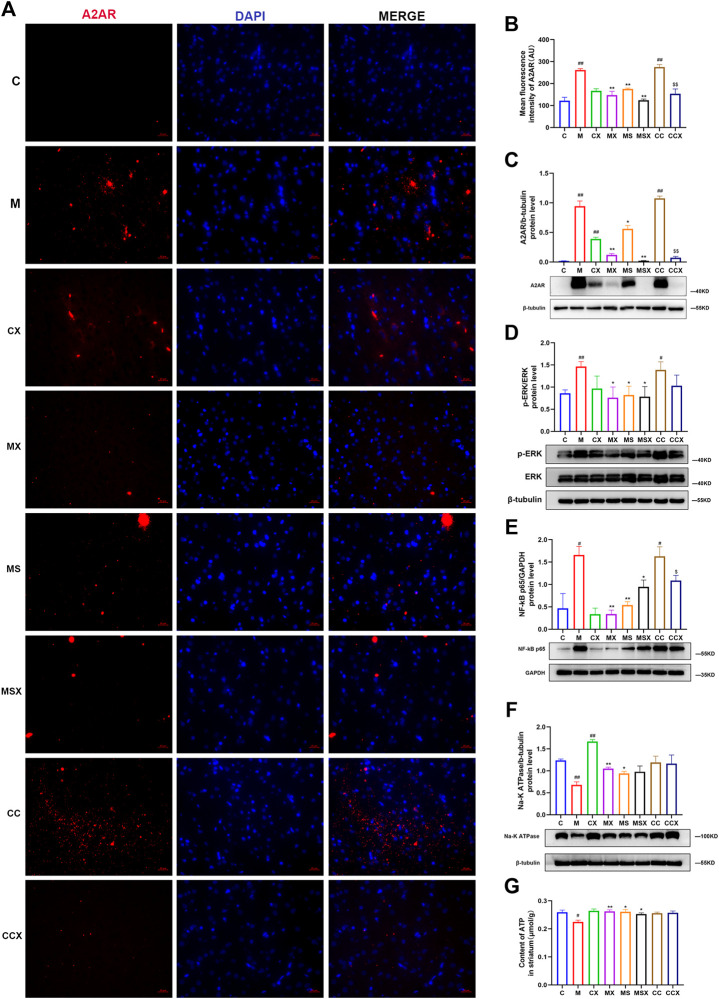
Effects of XYS on the striatal A2AR-ERK-NF-κB pathway. **(A)** Immunofluorescence staining of striatal A2AR (scale= 20 μm, 400× magnification). **(B)** Mean fluorescence density of A2AR. **(C)** The protein expression of A2AR in the rat striatum. **(D)** The protein expression of ERK in the rat striatum. **(E)** The protein expression of NF-κB p65 in the rat striatum. **(F)** The protein expression of NA-K ATPase in the rat striatum. **(G)** The ATP content in the rat striatum. The value is expressed as the mean ±SEM, *n* = 3. ^#^ *p* < 0.05 or ^##^ *p* < 0.01 versus the C group; ^*^ *p* < 0.05 or ^**^ *p* < 0.01 versus the M group; ^$^ *p* < 0.05 or ^$$^ *p* < 0.01 versus the CC group. A2AR, adenosine A2A receptor; ERK, extracellular signal-regulated kinase; NF-κB, nuclear factor kappa B; ATP, adenosine triphosphate.

As shown in Figures 5A–E, compared with those in the C group, the expression levels of A2AR (*p* < 0.01, *p* < 0.01), p-ERK (*p* < 0.05, *p* < 0.01), and NF-κB p65 (*p* < 0.05, *p* < 0.05) in the striatum of rats in the M group and CC group were higher. These increases in the protein expression levels were downregulated by XYS and SCH 58261 treatment (*p* < 0.05 or *p* < 0.05). XYS also decreased the protein expression levels of A2AR (*p* < 0.01), p-ERK (*p* < 0.05), and NF-κB p65 (*p* < 0.05) in the striatum of rats treated with the A2AR agonist.

As shown in Figures 5F,G, compared with the C group, the protein expression of NA-K ATPase (*p* < 0.01) and the contents of ATP (*p* < 0.05) in the rat striatum were decreased in the M group. XYS (*p* < 0.01, *p* < 0.01) or SCH 58261 (*p* < 0.05, *p* < 0.05) treatment increased these changes. The A2AR agonist did not change the protein expression of NA-K ATPase or the ATP content in the rat striatum compared with the C group (*p* > 0.05, *p* > 0.05).

Taken together, our results indicated that treatment with XYS could reverse the CRS-induced A2AR-ERK-NF-κB pathway in the rat striatum.

## Discussion

The purpose of this study was to investigate the effects of XYS on the depression-like phenotype of rats exposed to CRS, and to explore whether the potential mechanism is associated with A2AR signaling in the striatum. We found that XYS could alleviate the CRS-induced depression-like phenotype (such as body weight loss and depression- and anxiety-like behaviors) and played a protective role in the rat striatal synaptic ultrastructure. Furthermore, exposure to CRS and an A2AR agonist (CGS 21680) induced the activation of microglia and the A2AR-ERK-NF-κB pathway in the striatum, which was reversed by treatment with XYS.

XYS has been used to treat depression in China for thousands of years, and the clinical effect is remarkable. With the increasing attention of complementary alternative medicine, the safety and efficacy of XYS in treating depression have been recognized by many clinical researchers (Ding et al., 2020; Liu et al., 2020). Previous studies have indicated that XYS significantly improves anxiety-like behaviors and hippocampal neuron damage in stressed rats (Li et al., 2017). In this study, the results of behavioral tests indicated that treatment with XYS may mitigate CRS-induced depression-like phenotypes. Moreover, XYS also ameliorated the microstructural alterations in dendritic spine morphology and spine density in the rat striatum induced by CRS. Additionally, XYS is involved in the regulation of adenosine receptors, which are responsible for the beneficial biological effects against depression.

Clinical studies have indicated that morphological changes in the striatum can lead to a decrease in logical memory, recall delays and other psychiatric test scores (Madsen et al., 2010). In this study, microstructural alterations in dendritic spine morphology and spine density in the rat striatum caused by CRS were reversed by XYS. Moreover, XYS reduced the damage to the synaptic ultrastructure in the rat striatum induced by CRS and plays a protective role in the synaptic ultrastructure. In this study, XYS increased the protein expression of BDNF. BDNF is considered an instructive mediator of functional and structural plasticity in the central nervous system (Colucci-D'Amato et al., 2020). A2AR activation modulates several brain processes, ranging from neuronal maturation to synaptic plasticity. Most of these actions occur through the modulation of the actions of the BDNF (Ribeiro et al., 2021). A previous study reported that exercise alleviates depression-like behavior in chronically stressed rats by increasing BDNF in the striatum (Marais et al., 2009).

Microglial activation and neuroinflammation play very important roles in the process of depression (Troubat et al., 2021). Microglia can mediate some biological processes, such as abnormal secretion of toxic and health hazardous substances, removal of damaged cells and dysfunctional synapses through physical contact with biological neurons (Yirmiya et al., 2015). However, overactivation of microglia may result in the elimination and engulfment of functional synapses. This condition is known as “synaptic stripping”, and even leads to morphological changes in the brain (Kettenmann et al., 2013). In this study, the rats in the CRS group and A2AR agonist group showed increased Glu secretion and overactivation of microglia in the striatum, but XYS and A2AR antagonists reduced these changes. The abnormal release of Glu can quickly attract microglia to these brain regions, which then surround the overactive neurons (Eyo et al., 2014). This suggests that XYS may play a protective role in the synaptic ultrastructure by reducing the microglial activation. It is reported that microglia-mediated synaptic pruning can be mitigated by minocycline, an agent that reduces microglial activation (Jawaid et al., 2018).

A2AR is specifically enriched in the striatum and localized in glutamatergic synapses (Rebola et al., 2005) and glial cells (Matos et al., 2012). A2AR expression is usually low under physiological conditions but increases in the brain during stress. In terms of treatment, inhibition of A2AR can prevent neuroinflammation (Martí Navia et al., 2020). Therefore, adenosine A2AR antagonists are very important for the protection of the central nervous system. In this study, we found that XYS treatment downregulated the increases in the protein expression levels of A2AR in the striatum of rats induced by CRS. Studies have found that A2AR mediates phosphorylation and upregulation of the extracellular signal ERK1/2, resulting in increased Glu release (Braga et al., 2019; Köfalvi et al., 2020), causing high NF-κB expression and promoting microglial proliferation and activation (Mohamed et al., 2016). Our data also showed that the expression levels of p-ERK and NF-κB p65 in the striatum of rats in the CRS model group were increased, whereas XYS treatment downregulated these protein levels. The results above support our hypothesis and shows that XYS inhibits the A2AR-ERK-NF-κB pathway in the striatum, and this effect is similar to that of an A2AR antagonist.

We used a group of rats treated with CGS 21680 (an A2AR agonist) to investigate the effects of XYS on A2AR signaling in the striatum. Previous studies have suggested that A2AR serves an important role in the onset of microglial activation and inflammation after exposure to a low glucose/hypoxia in a NF-κB-dependent manner (Huang et al., 2018). We found that intraperitoneal injection of CGS 21680 can also activate the A2AR-ERK-NF-κB pathway in the striatum and induce a depression-like phenotype, which was reversed by treatment with XYS. These results further validated our hypothesis that the antidepressive effects of XYS are associated with A2AR signaling in the striatum.

There were several limitations of the current study. First, only male adult rats were included in the present study. As it has been pointed out in many studies that sex differences may affect the onset of depression, more investigations will be performed in the future. Second, we only used an A2AR antagonist to block the ERK-NF-κB pathway, but according to the A1R-A2AR imbalance theory, enhanced A1R also has an antidepressant effect (Serchov et al., 2015). Whether A1R agonists have similar effects requires further research.

## Conclusion

**FIGURE 6:**
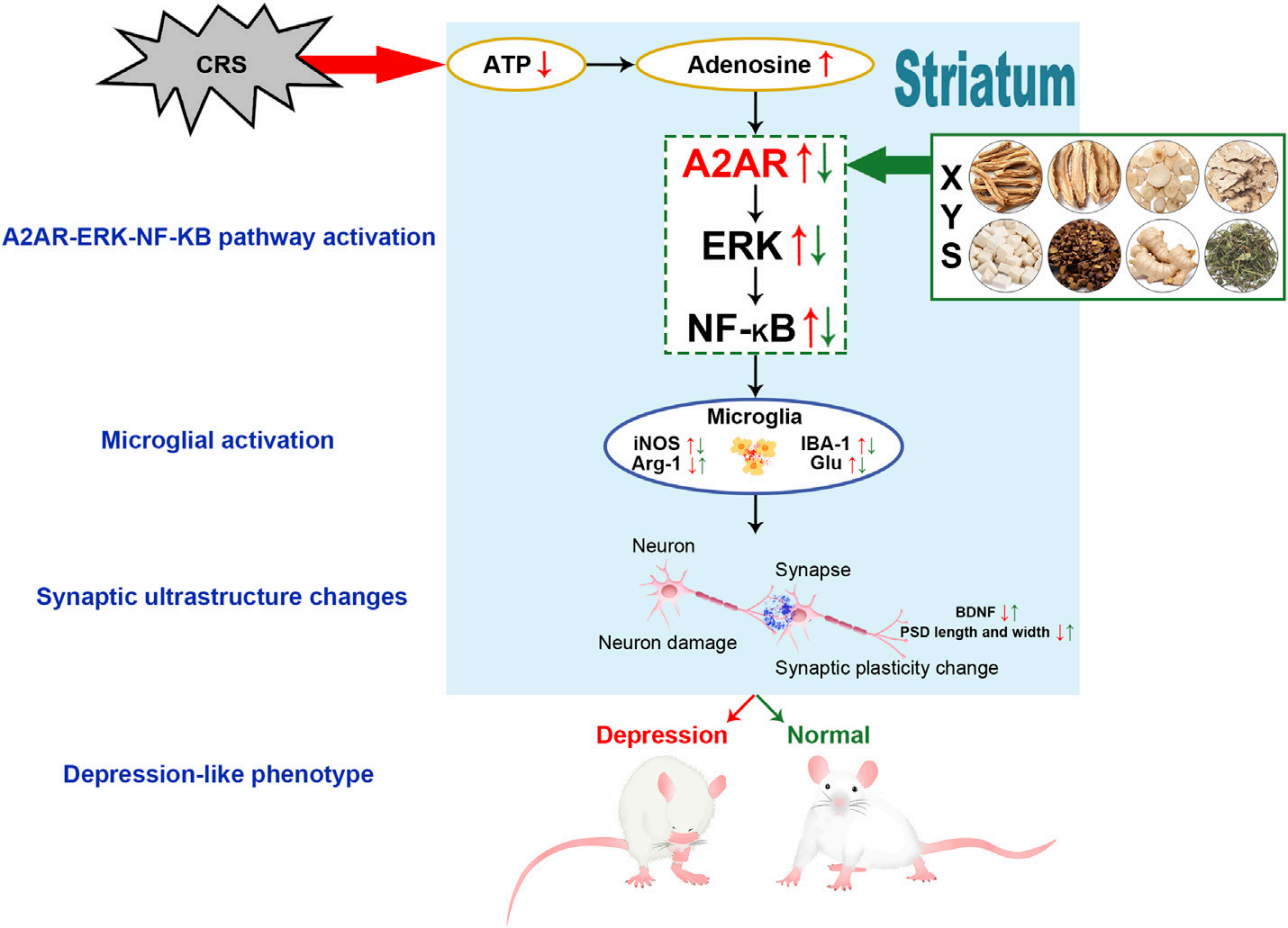
Potential mechanism by which XYS ameliorates the CRS-induced depression-like phenotype.

In summary, in this study, we found that XYS can ameliorate depression-like phenotypes and plays a protective role in the synaptic ultrastructure of the striatum. The mechanisms of these effects may be related to inhibition of the A2AR-ERK-NF-κB pathway in the striatum (Figure 6). Our findings serve as a pharmacological basis for the clinical application of XYS and provide a better understanding of the mechanisms associated with the regulatory effects of XYS on AR signaling.

## Data Availability

The original contributions presented in the study are included in the article/Supplementary Material, further inquiries can be directed to the corresponding authors.

## Abbreviations

CRS, chronic restraint stress; XYS, Xiaoyaosan; AR, adenosine receptor; A1R, adenosine A1 receptor; A2AR, adenosine A2A receptor; MEK, mitogen-activated protein kinase kinase; ERK, extracellular signal-regulated kinase; NF-κB, nuclear factor-κB; OFT, open field test; EPMT, elevated plus maze test; SPT, sucrose preference test; FST, forced swimming test; ATP, adenosine triphosphate; ATPase, adenosine triphosphatase; Glu, glutamic acid; WB, Western blot; BDNF, brain-derived neurotrophic factor; IBA-1, ionized calcium-binding protein-1; Arg-1, arginase-1; iNOS, inducible nitric oxide synthase; TNF-α, tumor necrosis factor-α; PSD, post-synaptic density.
